# Supplementary material for: Randomized controlled evaluation of the psychophysiological effects of social support stress management in healthy women
Source: PLoS One. 2021 Jun 4;16(6):e0252568. doi: 10.1371/journal.pone.0252568 (PMC8177426; doi:10.1371/journal.pone.0252568)
Supplement: S1 Appendix — (DOCX) [file pone.0252568.s002.docx]

Protocol of the proposed study

**Psychoneuroendocrine evaluation of social support stress management training in healthy women**

Prof. Dr. Jens Gaab

Division of Clinical Psychology and Psychotherapy

Department of Psychology

University of Basel

Missionsstrasse 62

4055 Basel

02.02.2015

Version 2

Basel, 02. February 2015

_____________________________________________________________

Prof. Dr. Jens Gaab

**Content**

1. Summary of the research plan 3
2. Research plan 4
   1. Current state of research 4
   2. Theoretical underpinning of proposed research plan 5
   3. Aims of the study 5
3. Methods 6
   1. Study design and data analysis 6
   2. Data analysis and sample size estimation 6
   3. Recruitment and randomisation 7
   4. Inclusion and exclusion criteria 8
   5. Explanations of the used variables 8
4. Duties of the investigator and quality management 11
5. Ethical Aspects and Quality Assurance 11
6. Documentation of the data, quality control and quality assurance 12

6.1 Budget 12

1. Signature of the principal investigators 12
2. References 13
3. Attachment: Plan of the social support intervention 17
4. **Summary of the research plan**

Psychosocial stress is a potent activator of the hypothalamus–pituitary–adrenal (HPA) axis. While neuroendocrine stress responses are essential for the maintenance of homeostasis, evidence suggests that excessive activation of the HPA axis constitutes a risk for disease and psychopathology. Previously, our group has shown that cognitive-behavioural as well as resource-focussed stress management trainings effectively reduce the activation of the HPA axis under stress in healthy subjects. However, these interventions focus on fight-or-flight type responses to stress.

Numerous studies demonstrated the essential importance of interpersonal ties, social support and social integration for health, wellbeing and even survival, which are mediated to a large extent by direct effects of social support on biological parameters of stress reactivity, including autonomous nervous system markers as well as immune and neuroendocrine markers. Although the positive effects of social support are well supported as well as long accepted, a stress management intervention based on social support has not been designed and tested so far.

Therefore, the purpose of the present study is to develop and evaluate the effects of social support-based stress management training on psychological wellbeing and endocrine stress responses under acute psychosocial stress among healthy young subjects.

1. **Research plan**

***2.1 Current state of research***

Psychosocial stress can be conceptualized as incongruence between the abilities and needs of a person and the environment. At the individual level, psychosocial stress is the result of a cognitive appraisal of what is at stake and what can be done about it (Lazarus, 2005; Lazarus & Folkman, 1984). It is assumed that the result of this subjective appraisal influences the emotional, behavioural, and biological responses to stress.

Regarding biological responses to psychosocial stress, hormones of the hypothalamus pituitary adrenal (HPA) axis have been examined extensively. Although the activation of the HPA axis in response to stress and its effects on cardiovascular, immune, and metabolic as well as emotional, behaviour, and cognitive processes helps to maintain equilibrium during and after stress (Sapolsky, Romero, & Munck, 2000), extensive and/or long- standing release of HPA axis hormones has been shown to have detrimental effects on somatic and emotional well-being (McEwen, 1998; Seeman, McEwen, Rowe, & Singer, 2001). Beside genetic (Wüst et al., 2004), endocrine (Kirschbaum, Kudielka, Gaab, Schommer, & Hellhammer, 1999), and habitual (Kirschbaum, Wüst, & Strasburger, 1992) factors, situation-specific anticipatory cognitive appraisal, but not general personality variables, substantially account for the extent of the biological response in the face of stress (Gaab, Rohleder, Nater, & Ehlert, 2005). Particularly the perception of threat to the social self that is judged as uncontrollable is a major situational trigger of the HPA axis activation in response to stress (Dickerson & Kemeny, 2004).

While uncontrollable social evaluation was shown to be one of the strongest stressors (Dickerson & Kemeny, 2004), positive social interactions are essential to physiological and psychological wellbeing. Being integrated in close social relationships or perceiving that social support would be available in case of need has considerable positive consequences for health and even survival (Holt-Lunstad, Smith, & Layton, 2010), with effect sizes equalling or exceeding those of well-established behavioural factors such as smoking cessation, exercise, and abstinence from alcohol. Both the direct positive effect of social support and integration on various health parameters as well as the reduction of the incidence or frequency of stress are discussed as putative mechanisms of stress-protective effects by social support (Cohen & Wills, 1985; Cohen, 1988).

Beside the long-term health outcomes, direct effects of social support on acute stress reaction were intensively investigated in the last decades. Instructed social support reduces autonomic nervous system activation to stress measured on cardiovascular parameters. This effect is moderated by quality of the interaction, and was shown to be the strongest for non-evaluative positive emotional support (Allen, 2002; Gerin, Pieper, Levy, & Pickering, 1992; Kamarck, Manuck, & Jennings, 1990; Lepore, Allen, & Evans, 1993). Similarly, reduction of endocrine stress response (lower salivary free cortisol levels) to a standardized laboratory stress test TSST was observed both for men and women (Kirschbaum, Pirke, & Hellhammer, 1993). Interestingly, men benefited more from verbal support by their partner, whereas women from non-evaluative nonverbal support expressed in touch, hugs or smiling, while the verbal support by their partner rather increased the cortisol levels instead (Ditzen et al., 2007; Heinrichs, Baumgartner, Kirschbaum, & Ehlert, 2003).

Taylor et al. (2000) postulated that the stress-protective effects of social support are differently utilized by men and women. Beside the fight-or-flight behaviour that may characterize the primary physiological response to stress for both males and females, behaviourally, females’ responses are more marked by a pattern of tend-and-befriend. Tending is defined as nurturing activities to protect the self and offspring, promote safety and reduce distress; and befriending as the creation and maintenance of social networks that may aid in this process. In accord to this hypothesis, interpersonal stressors like social rejection elicited higher free cortisol increases in women that in men, whereas situations involving intellectual inferiority and performance failures were perceived as more stressful by men (Stroud, Salovey & Epel, 2002).

- 1. ***Theoretical underpinning of proposed research plan***

A number of studies have shown that cognitive–behavioural stress management (CBSM) effectively reduces basal cortisol levels and/or psychological morbidity shortly after the intervention in clinical as well as nonclinical populations (HIV: Berger et al., 2008, Antoni et al., 2000, and Cruess et al., 1999; breast cancer: Cruess et al., 2000; athletes: Perna, Antoni, Kumar, Cruess, & Schneiderman, 1998). Also, in healthy participants, CBSM was observed to attenuate cortisol responses to a standardized stress test 2 weeks as well as 4 months after training (Gaab et al., 2003; Hammerfald et al., 2006).

The mentioned interventions aim to address explicit levels of self-evaluation. Individuals reflect on their experiences, evaluate the contents of consciousness, and introspect about the causes and meanings of stressful situations.

In most cases the studies were conducted with only male participants, to exclude the methodological difficulty that the intensity of endocrine response in females of reproductive age depends on the phase of the cycle and use of oral contraceptives (Kirschbaum et.al., 1999). The CBSM intervention was effective for men (Gaab et al., 2003), and when both sexes were participating, the effect of cognitive training for women was weaker (Hammerfald et al., 2006). Thus, it can be speculated that these types of stress management address rather the fight-or-flight response and might be in general more beneficial for men that for women.

Previous research indicates the essential direct effects of social support on stress reactivity and the presumably higher importance of interpersonal processes for women. The goal of this study is to develop and evaluate a tend-and-befriend type stress management intervention. Since we hypothesize, that social support intervention targets the postulated, primarily feminine, tend and befriend stress coping mechanism, its efficacy will be tested on female subjects.

***2.3 Aims of the study***

In summary, the majority of studies on the effects of stress management training are based on a problem-oriented fight-or-flight type of stress coping. The goal of this study is to develop a tend-and-befriend based stress management intervention and test its effects on acute neuroendocrine, psychophysioloigcal as well as subjective stress responses as well as on psychological well-being, using a methodological approach similar to that used in previous CBSM studies (Gaab et al., 2003; Hammerfald et al., 2006, Storch et al., 2007).

1. **Methods**

***3.1 Study design and data analysis***

This study is an experimental study with parallel groups. Subjects are randomized into the two conditions: Treatment condition, thus receiving tend-and-befriend training (TBT) *before* the TSST; control conditions = waitlist controls receiving tend-and-befriend training (TBT) after the termination of the study. Two weeks after the participants of the treatment condition have received the tend-and-befriend training (TBT), all participants of the treatment as well as the control condition will undergo a standardized psychosocial stress test, the Trier Social Stress Test (TSST, Kirschbaum et al., 1993). The study variables are displayed in Table 1, the flow through the study is shown in Table 2.

Table 1. Study variables

| Variables | Description | Time |
| --- | --- | --- |
| Independent Variable | Tend-and-befriend training (TBT) and waitlist control condition (WL-CC) | TBT two weeks before TSST, WL-CC after termination of study |
| Primary outcome | Cortisol and alpha-amylase | 9 measurements before, during and after TSST |
| Secondary outcomes | Psychophysiological parameters (heart rate, electrodermal activity, electrocardiography, electromyography, respiratory activity and skin temperature) | Repeated assessment before, during and after TSST |
|  | Questionnaires (PASA, STAI X-1, STAI X-2, PSS, FSozU, BFW/E, ASS-SYM) | TSST: PASA, STAI X-1  Baseline, post and follow-up: STAI X-2, PSS, FSozU, BFW/E, ASS-SYM |

***3.2 Data analysis and sample size estimation***

Multivariate analyses of covariance and variance for repeated measures will be computed to analyse endocrine and psychophysiological stress responses as well as pre-post changes of psychometric scores between groups, controlling for differences in baseline levels when indicated. Regression analysis will be used to assess associations between psychological and cortisol parameters. For cortisol and psychophysiological parameters, areas under the response curve will be calculated with respect to increase (AUCi) and ground (AUCg), using the trapezoidal method as an indicator for the integrated biological and physiological response in the TSST (Pruessner, Kirschbaum, Meinlschmid, & Hellhammer, 2003). Data will be tested for normal distribution and homogeneity of variance using the Kolmogorov-Smirnov test and Levene’s test before statistical procedures were applied. Based on previous own studies (Gaab et al., 2003, Hammerfald et al., 2006, Storch et al., 2007), we expect a large multivariate effect size (f^2^=0.35), Therefore, the optimal total sample size of N = 48 to detect an expected large effect size of f2 = 0.35 (representing a large effect size) with a power = 0.85 and α = 0.05 was calculated a priori with the statistical software G-Power (Buchner et al., 1997). For all analyses, significance level is α = 5%. All analyses are performed according to the principle of intention to treat, with last observations carried forward when follow-up data were missing.

*Table 2: Flow through the study*

|  | TBT | WL-CC | |
| --- | --- | --- | --- |
| Baseline assessment | Advertisement with bulletins and BAPS  Information about study procedure on BAPS, screening for inclusion- and exclusion criteria on BAPS  STAI X-2, PSS, FSozU, BFW/E, ASS-SYM | | |
| Intervention | TBT | | - |
| Stress test | TSST  Assessment of 9 saliva samples -30, -20, -10, 0, 10, 20, 30, 45 and 60 min (with 0 min being directly post-TSST) for salivary free cortisol and alpha-amylase.  Continuous assessment of psychophysiological parameters (heart rate, electrodermal activity, electrocardiography, electromyography, respiratory activity and skin temperature)  Questionnaires: PASA (once), STAI X-1 | | |
| Post assessment | STAI X-2, PSS, FSozU, BFW/E, ASS-SYM | | |
| Follow-up assessment | STAI X-2, PSS, FSozU, BFW/E, ASS-SYM | | |

***3.3 Recruitment and randomisation***

Participants will be recruited via the online recruitment system of the Department of Psychology (BAPS-Sona, http://baps.sona-systems.com) and in lectures at the University of Basel. Subjects interested in participating in the study can sign up by e-mail. All interested subjects will be screened for inclusion and exclusion criteria and be informed verbally about the goal and the procedure of the study by telephone interview. In case of willingness to participate in the study, subjects receive a written description of the study aims, theoretical background as well as employed psychotherapeutic methods and experimental procedures (i.e. randomization and TSST) as well as the written informed consent and the a link to the baseline assessments (see below).

Upon receipt of the signed written informed consent and the filled-out questionnaires, participants will be randomized through individual assignment codes in properly concealed between black sheets, stored in sequentially numbered envelopes. After randomization, participants will be informed about study dates, thus dates for the tend-and-befriend training (TBT) and the TSST. The study participation is voluntary. Subjects can end their participation anytime and without any reason and disadvantage. The participation can be ended by the study staff when participation might have detrimental effects (e.g. participants appears to be in psychosocial crisis), the study design is not followed and in case of adverse events. The participation in the study must be ended if in retrospect the inclusion criteria do not apply to the subject or if one exclusion criterion is met.

***3.4 Inclusion and exclusion criteria***

Inclusion criteria are being between 18 and 60 years old, female gender and healthy by self-report statement, thus no known current or chronic somatic diseases or psychiatric disorders. Exclusion criteria are male gender, any acute or chronic disease (chronic pain, hypertension, heart disease, renal disease, liver disease, diabetes) as well as skin pathologies, neuropathies or nerve entrapment symptoms, sensory abnormalities affecting the tactile or thermal modality, current medications (psychoactive medication, narcotics, intake of analgesics) or being currently in psychological or psychiatric treatment, insufficient German language skills to understand the instructions, previous participation in studies using the TSST and daily consumption of more than three alcoholic standard beverages per day (a standard alcoholic beverage is defined as either 3dl beer or 1 dl wine or 2cl spirits).

***3.5 Explanations of the used variables***

***Independent Variable:*** *tend-and-befriend training (TBT)*

Participants will attend a group-based tend-and-befriend training (TBT). The intervention will be conducted in group format, led by two supervised master students. The intervention will first address the rational of the intervention, thus the psychobiological effects of stress, the importance of the social support and integration for health and well-being. The main stake of the intervention is devoted to small group-based exercises to promote social and emotional intelligence of the participants, improve interpersonal communication to improve the quality of their interpersonal interactions, and give room for personal and interpersonal exploration. During the intervention participants are encouraged to build new supportive relationships and to strengthen or revive existing interpersonal ties to both receive and give their support to others. Finally, scenarios of acute and chronic stress situations will be discussed, and the participants will train to adopt more social support based strategies in their coping repertoire. The participants will be encouraged to practice the skills with each other or other close relations between the sessions. For the intervention’s detailed description, please see Attachment 1. Since the intensity of endocrine response in females depends on the hormonal phase and the use of oral contraceptives (Kirschbaum et al., 1999), this information will be collected directly before the TSST and considered by the test evaluation.

***Dependent Variables:***

*Trier Social Stress Test (TSST)*

The Trier Social Stress Test (TSST) has repeatedly been found to induce profound endocrine and cardiovascular responses in 70–80% of the subjects tested (Kirschbaum et al., 1993). After a basal sample of salivary free cortisol and the completion of the STAI-State, subjects are introduced to the TSST (two minutes). They then return to a different room, where they have ten minutes to prepare and to complete a questionnaire designed to assess cognitive appraisal processes (PASA, see below) regarding the anticipated stress situation as well as the second STAI-State. Afterwards, subjects are led back into the TSST room, where they take part in a simulated job interview (five minutes) followed by a mental arithmetic task (five minutes) in front of an audience of two people. To assess salivary free cortisol levels and alpha-amylase 9 saliva samples are taken 30, 20 10 minutes before and immediately after the TSST – at 0, 10, 20, 30, 45, and 60 minutes. Also, the STAI-state is assessed directly after the TSST as well as with the last saliva sample at 60 min post-TSST. The TSST will be performed from 14.00h to 18.00h. So far, the TSST has been considered the gold standard for stress measurements in laboratory settings due to its reliability, validity and standardization (Dickerson and Kemeny, 2004). The TSST has already been applied in many studies to different healthy and clinical populations as well as populations with somatic and psychiatric disorders. Negative short-term and long-term effects of the TSST have not been observed so far. All TSST participants get a full debriefing after the completion of the TSST, informing about its intentions the people involved in the test. The TSST has been approved by several Swiss ethical committees (Zurich, St. Gallen, Bern, Basel).

*Physiological parameters*

The impact of the social support based stress management training on physiological stress responses, cortisol levels as well as alpha-amylase activity will be assessed repeatedly before and after the TSST. Cortisol is the most important and easy to measure parameter of the HPA activity (Kirschbaum & Hellhammer, 1994). Saliva sample are to be collected through the Salivette system at 9 measurement times (30, 20,10 minutes before the TSST as well as 0, 10, 20, 30, 45, 60 minutes after the end of the TSST). The participants chew on a small cotton roll for about 1 minute, which afterwards is put into a small plastic container (Kirschbaum & Hellhammer, 1994). This easy and non-invasive technique is a reliable and valid solution to measure cortisol and amylase concentrations in the saliva. The Salivette is frozen at -20°C immediately after the sample collection. After thawing, the following biochemical analyses were conducted in the biochemical laboratory of the Clinical Psychology and Psychotherapy department at the University of Zurich, Switzerland: For salivary cortisol analyses a highly sensitive liquid chromatography–tandem mass spectrometry (LC–MS/MS) method was used and the activity of salivary alpha-amylase (sAA) was analyzed with a kinetic colorimetric test using assay kits (Roche 11555685 alpha-Amylase Liquid acc) and the automatic analyzer (Bio- tek Instruments, Lucern, Switzerland) with software KC4 (Roche, Basel, Switzerland). After analysis, saliva samples will be stored for 1 year at -20°C.

All psychophysiological parameters assessed are part of the wireless physiological recording system BioNomadix (Biopac Systems, Inc., Santa Barbara, CA 93117). Wireless, wearable physiology monitoring devices for life science research that noninvasively record high quality data while comfortably allowing subjects to move freely will be used. BioNomadix devices use digital transmission and short leads placed close to the signal source to provide optimal signal quality. The BioNomadix transmitter unit uses short leads for easy placement at any point of interest and is easily attached to the body with soft Velcro straps. Psychophysiological data is imported, edited, and analyzed using the AcqKnowledge 4.3 software for Windows. For assessment of the respective parameter, see below:

- Heart frequency: Infrared emitter and photo-diode transmit changes in infrared reflectance from varying blood flow. The lead is either placed on the subject’s ear or on a finger of the nondominant hand.
- EDA: Skin conductance is recorded using exosomatic method with Direct Current. Two Biopac electrodes are attached at distal phalanges of two fingers of the subject’s nondominant hand
- ECG (2-lead): Two lead electrocardiographic activity is recorded with three disposable, pregelled electrodes attached to the skin.
- EMG: Two electrodes are placed at the upper trapezius muscle to assess its activation according to former stress studies
- Respiratory rate: With a chest belt changes in thoracic or abdominal circumference as subject breathes are observed.
- Skin temperature: Stainless stell banjo design (taped to body).

*Psychological variables*

All psychological variables will be assessed online and coded, i.e. will not contain any names or birthdays, which could allow identification of participants.

- The *Primary Appraisal Secondary Appraisal Scale* (PASA, Gaab et al., 2005) is specifically constructed to assess cognitive appraisal processes in the TSST according to transactional stress theory. The PASA is composed of four situation-specific subscales assessing (primary appraisal) Challenge and Perceived Threat as well as (secondary appraisal) Self-Concept of One’s Own Competence and Control Expectancy. The primary scales can be summarized to form two secondary scales (Primary Appraisal and Secondary Appraisal). Scales range from 1 (*very little*) to 6 (*very much*). To be able to assess anticipatory cognitive appraisals, the PASA is administered between the introduction to the TSST and the actual TSST.
- *The State-Trait Anxiety Inventory* (STAI, [Laux, Glanzmann, Schaffner, & Spielberger, 1981](#_ENREF_22)): This instrument assess not only state but also trait anxiety. The STAI X-1 consists of 20 statements and the answers to these are used to judge a patient's degree of anxiety at a specific time (state anxiety) and is completed prior to the introduction to the TSST, just before, and 1 hour after the TSST. The STAI X-2 form consists of a different set of 20 statements, and the answers to these are used to calculate a patient's underlying (trait/personality) degree of anxiety. STAI X-2 is administered at the baseline assessment, directly after the intervention and at the follow-up assessment, 4 weeks after the intervention. Each statement in both STAI is rated on a 4-point scale according to the patient's agreement with that statement. The overall score for STAI ranges from a minimum of 20 to a maximum of 80; STAI scores are commonly classified as “little or no anxiety” (20–37), “moderate anxiety” (38–44), and “extreme anxiety” (45–80). The duration is defined as 3 to 6 minutes per scale.
- A German translation of the *Perceived Stress Scale* (PSS, Cohen et al., 1983) will be used to assess the degree to which situations experienced during the last few days are perceived as stressful. Items in the PSS are designed to assess how predictable, uncontrollable, and overloading participants find their lives at the moment. The PSS is administered at the baseline assessment, directly after the intervention and at the follow-up assessment, 4 weeks after the intervention.
- *Fragebogen zur Sozialen Unterstützung (*ll, Fydrich, Sommer & Brähler, 2007, 22 items version) will be used to assess the subjective availability of resources from the participants’ social network. The questionnaire covers following dimensions (1) emotional support, (2) practical support, (3) social integration, (4) burden coming from social network, as well as (5) reciprocity, (6) availability of a trusted person, and (7) satisfaction with the social support. Each statement is rated on a 5-point scale according to the patient's agreement with that statement. The FSozU is administered at the baseline assessment, directly after the intervention and at the follow-up assessment.
- *Berner Fragebogen zum Wohlbefinden (BFW/E, Grob, 1995*) with 39 items will be used to assess the subjective wellbeing. The items cover positive attitude to life, self-esteem, depressive mood, problem awareness, somatic symptoms and zest for life. The questionnaire takes 5-10 minutes to complete.
- With the change-sensitive symptom list concerning relaxation experience, well-being, discomfort and preoccupation level (ASS-SYM), six indication and effect ranges of systematic relaxation methods are covered by 48 items concerning discomfort and preoccupation level. The items are comprehensive and quickly answered and cover generally significant indicators about well-being, relaxation experience, discomfort and preoccupation level. Subscales (with 8 items each) refer to: (1) physical and psychological exhaustion, (2) nervousness and inner tension, (3) psychophysiological deregulation, (4) performance and behavioural problems, (5) burden of pain, (6) problems with self-determination and –control. Additionally an overall scale (ASS-SYM-G) referring to the general level of symptoms and problems is administered. It takes 10 to 15 minutes to complete the symptom list.

1. **Duties of the investigator and quality management**

All personal data will be safely handled according to this protocol, the guidelines of good clinical practice and to legal regulations. The subject’s data will be coded. A code will be attributed to each subject and will be saved in digital form. The codes can be provided to the Ethics Committee at any time. Only the scientific collaborators involved in this study will have access to the collected data.

The occurrence of possible undesirable events (UE) will be assessed by email after the training and by interview after the TSST with each participant with an open question. Any answer will be recorded in the CRF. An UE is defined as an adverse or accidental medical or psychological reaction. All UE will be reported to the primary investigator (JG) within 24h after the assessment. In the event of study discontinuation the Ethics Committee will be informed according to the Art 22 HFV.

For the planned study, the subjects will be protected by insurance. Application for insurance proposal is in process.

1. **Ethical aspects and Quality Assurance**

*Protection of privacy*

All personal data will be handled securely according to Swiss Data Protection Regulation. The subject’s data will be coded. A code will be attributed to each subject and will be saved in digital form. The codes can be provided to the Ethics Committee at any time. Only the scientific staff involved in this study will have access to the collected data.

*Direct consequences for the involved participants*

This study has to be considered as basic research with pure scientific interest. The study is of no direct benefit for the volunteers. Participants receive either a financial (CHF 50) or administrative (5 "Versuchspersonenstunden" as part of their Bachelor studies) reimbursement.

The participants will be exposed to the TSST, which is an unpleasant and stressful situation. However, the TSST has been used in different healthy and patient population and so far, no intermediate or long-term negative consequences have been published. Also, the TSST has been used at the Department of Psychology and by the primary investigator (JG) many times. Additionally and most importantly, volunteers can stop TSST themselves at any time during each experimental run, without negative consequences. Therefore, the risks for the participants are considered as low. The benefit of the study is that participants receive tend-and-befriend training (TBT) at no costs. Also, participants will repeatedly be questioned about their personality traits and well-being with different questionnaires. There are no known negative effects of that kind of interrogation. The participants are informed about the whole procedure and potential risks. A written consent will also be signed before entering the study. The written consent explains the goals and risks of the study and clarifies that the participants have the right to end the study whenever they want.

1. **Documentation of the data, quality control and quality assurance**

The original data concerning the study are accessible for audits of the respective ethics committee. These inspections are conducted according to the legal conditions of data protection. The case report forms contain information about every single subject participating in the study. Data will be stored at the Department of Psychology for 10 years.

*6.1 Budget*

All direct and indirect costs involved (cortisol and alpha amylase assays: CHF 4500, reimbursement of participants: CHF 2500 as well as any allowances) will be covered by the Division of Clinical Psychology and Psychotherapy, Department of Psychology, University of Basel.

1. **Signature of the principal investigator**

Basel, 2.2.2015

Prof. Dr. Jens Gaab

1. **References**

Allen, K. (2002). Cardiovascular Reactivity and the Presence of Pets, Friends, and Spouses: The Truth About Cats and Dogs. *Psychosomatic Medicine*, *64*(5), 727–739.

Antoni, M. H., Cruess, S., Cruess, D. G., Kumar, M., Lutgendorf, S., Ironson, G., et al. (2000). Cognitive-behavioral stress management reduces distress and 24-hour urinary free cortisol output among symptomatic HIV-infected gay men. *Annals* *of* *Behavioral* *Medicine,* *22*(1), 29 –37.

Berger, S., Schad, T., von Wyl, V., Ehlert, U., Zellweger, C., Furrer, H., Regli, D., Vernazza, P., Ledergerber, B., Battegay, M., Weber, R. & Gaab, J. (2008). Effects of cognitive behavioral stress management on HIV-1RNA, CD4 cell counts and psychosocial Parameters of HIV infected persons. *AIDS, 22,* 767-775.

Bond, F. W., Hayes, S. C., Baer, R. A., Carpenter, K. M., Guenole, N., Orcutt, H. K., Waltz, T., Zettle, R. D. (2011). Preliminary psychometric properties of the Acceptance and Action Questionnaire-II : a revised measure of psychological inflexibility and experiential avoidance. *Behavioral Therapy, 42*(2), 672-688.

Buchner, A., Faul, F., & Erdfelder, E. (1997). *G*􏰂*Power:* *A* *priori,* *post-hoc,* *and* *compromise* *power* *analyses* *for* *the* *Macintosh* (Version 2.1.2). Trier, Germany: University of Trier.

Cohen, S., Kamarck, T., & Mermelstein, R. (1983). A global measure of perceived stress. *Journal* *of* *Health* *and* *Social* *Behavior,* *24*(4), 385–396.

Cohen, S. (1988). Psychosocial models of the role of social support in the etiology of physical disease. *Health Psychology : Official Journal of the Division of Health Psychology, American Psychological Association*, *7*, 269–297.

Cohen, S., & Wills, T. A. (1985). Stress, social support, and the buffering hypothesis. *Psychological Bulletin*, *98*(2), 310–57.

Cruess, D. G., Antoni, M. H., Kumar, M., Ironson, G., McCabe, P., Fernandez, J. B., et al. (1999). Cognitive-behavioral stress management buffers decreases in dehydroepiandrosterone sulfate (DHEA-S) and increases in the cortisol/DHEA-S ratio and reduces mood disturbance and perceived stress among HIV-seropositive men. *Psychoneuroendocrinology,* *24,* 537–549.

Cruess, D. G., Antoni, M. H., McGregor, B. A., Kilbourn, K. M., Boyers, A. E., Alferi, S. M., et al. (2000). Cognitive-behavioral stress manage- ment reduces serum cortisol by enhancing benefit finding among women being treated for early stage breast cancer. *Psychosomatic* *Medicine,* *62*(3), 304 –308.

Dickerson, S. S., & Kemeny, M. E. (2004). Acute stressors and cortisol responses: a theoretical integration and synthesis of laboratory research. *Psychological Bulletin, 130*(3), 355-391.

Ditzen, B., Neumann, I. D., Bodenmann, G., von Dawans, B., Turner, R. A., Ehlert, U., & Heinrichs, M. (2007). Effects of different kinds of couple interaction on cortisol and heart rate responses to stress in women. *Psychoneuroendocrinology*, *32*(5), 565–74.

Flaxman, P. E., & Bond, F. W. (2010). Worksite stress management training: moderated effects and clinical significance. *Journal of Occupational Health Psychology, 15*(4), 357-358.

Gaab, J., Blattler, N., Menzi, T., Pabst, B., Stoyer, S., & Ehlert, U. (2003). Randomized controlled evaluation of the effects of cognitive-behavioral stress management on cortisol responses to acute stress in healthy subjects. *Psychoneuroendocrinology,* *28,* 767–779.

Gaab, J., Rohleder, N., Nater, U. M., & Ehlert, U. (2005). Psychological determinants of the cortisol stress response: the role of anticipatory cognitive appraisal. *Psychoneuroendocrinology, 30*(6), 599-610.

Gerin, W., Pieper, C., Levy, R., & Pickering, T. G. (1992). Social support in social interaction: a moderator of cardiovascular reactivity. *Psychosomatic Medicine*, *54*(3), 324–336.

Hammerfald, K., Eberle, C., Grau, M., Kinsperger, A., Zimmermann, A., Ehlert, U., et al. (2006). Persistent effects of cognitive-behavioral stress management on cortisol responses to acute stress in healthy subjects—A randomized controlled trial. *Psychoneuroendocrinology,* *31,* *333*–339.

Heinrichs, M., Baumgartner, T., Kirschbaum, C., & Ehlert, U. (2003). Social support and oxytocin interact to suppress cortisol and subjective responses to psychosocial stress. *Biological Psychiatry*, *54*, 1389–1398. doi:10.1016/S0006-3223(03)00465-7

Holt-Lunstad, J., Smith, T. B., & Layton, J. B. (2010). Social relationships and mortality risk: a meta-analytic review. *PLoS Medicine*, *7*(7), e1000316.

Kamarck, T. W., Manuck, S. B., & Jennings, J. R. (1990). Social support reduces cardiovascular reactivity to psychological challenge: a laboratory model. *Psychosomatic Medicine*, *52*(1), 42–58.

Kirschbaum, C., Hellhammer, D.H., (1994). Salivary cortisol in psychoneuroendocrine research: recent developments and applications. *Psychoneuroendocrinology,* *19*, 313–333.

Kirschbaum, C., Kudielka, B. M., Gaab, J., Schommer, N. C., & Hellhammer, D. H. (1999). Impact of gender, menstrual cycle phase, and oral contraceptives on the activity of the hypothalamus-pituitary-adrenal axis. *Psychosomatic Medicine, 61*(2), 154-162.

Kirschbaum, C., Pirke, K. M., & Hellhammer, D. H. (1993). The “Trier Social Stress Test”—A tool for investigating psychobiological stress responses in a laboratory setting. *Neuropsychobiology,* *28*(1–2), 76 – 81.

Kirschbaum, C., Wüst, S., Faig, H. G., & Hellhammer, D. H. (1992). Heritability of cortisol responses to human corticotropin-releasing hormone, ergometry, and psychological stress in humans. *Journal of Clinical Endocrinology & Metabolism, 75*(6), 1526-1530.

Kirschbaum, C., Wüst, S., & Strasburger, C. J. (1992). “Normal” cigarette smoking increases free cortisol in habitual smokers. *Life* *Sciences,* *50*(6), 435– 442.

Kivimäki, M., Vahtera, J., Elovainio, M., Helenius, H., Singh-Manoux, A., & Pentti, J. (2005). Optimism and pessimism as predictors of change in health after death or onset of severe illness in family. *Health Psychology, 24*(4), 413-421.

Laux, L., Glanzmann, P., Schaffner, P. & Spielberger, C.D. (1981). Das State-Trait-Angstinventar. Theoretische Grundlagen und Handanweisung. Weinheim: Beltz Test GmbH.

Lazarus, R. S., & Folkman, S. (1984). *Stress,* *appraisal,* *and* *coping.* New York: Springer.

Lazarus, R. S. (2005). Emotions and interpersonal relationships: toward a person-centered conceptualization of emotions and coping. *Journal of Personality, 74*(1), 9-46.

Lepore, S. J., Allen, K. a, & Evans, G. W. (1993). Social support lowers cardiovascular reactivity to an acute stressor. *Psychosomatic Medicine*, *55*(6), 518–524.

McEwen, B. S. (1998). Protective and damaging effects of stress mediators. *New England Journal of Medicine, 338*(3), 171-179.

Perna, F. M., Antoni, M. H., Kumar, M., Cruess, D. G., & Schneiderman, N. (1998). Cognitive-behavioral intervention effects on mood and cortisol during exercise training. *Annals* *of* *Behavioral* *Medicine,* *20*(2), 92–98.

Penedo, F. J., Dahn, J. R., Gonzalez, J. S., Molton, I., Carver, C. S., Antoni, M. H., et al. (2003). Perceived stress management skill mediates the relationship between optimism and positive mood following radical prostatectomy. *Health Psychology, 22*(2), 220-222.

Pruessner, J. C., Kirschbaum, C., Meinlschmid, G., & Hellhammer, D. H. (2003). Two formulas for computation of the area under the curve represent measures of total hormone concentration versus time- dependent change. *Psychoneuroendocrinology,* *28,* 916 –931.

Richman, L. S., Kubzansky, L., Maselko, J., Kawachi, I., Choo, P., & Bauer, M. (2005). Positive emotion and health: Going beyond the negative. *Health* *Psychology,* *24,* 422– 429. Bern: Huber.

Sapolsky, R. M., Romero, L. M., & Munck, A. U. (2000). How do glucocorticoids influence stress responses? Integrating permissive, suppressive, stimulatory, and preparative actions. *Endocrine Reviews, 21*(1), 55-89.

Seeman, T. E., McEwen, B. S., Rowe, J. W., & Singer, B. H. (2001). Allostatic load as a marker of cumulative biological risk: MacArthur studies of successful aging. *Proceedings of the National Academy of Science, 98*(8), 4770-4775.

Storch, M., Gaab, J., Kuttel, Y., Stussi, A. C., & Fend, H. (2007). Psychoneuroendocrine effects of resource-activating stress management training. *Health Psychology, 26*(4), 456-463.

Stroud L., Salovey P., & Epel E. (2002). Sex differences in stress responses: social rejection versus achievement stress. *Biological Psychiatry, 52(4),* 318–327.

Taylor, S. E., Klein, L. C., Lewis, B. P., Gruenewald, T. L., Gurung, R. A., & Updegraff, J. A. (2000). Biobehavioral responses to stress in females: tend-and-befriend, not fight-or-flight. *Psychological Review*, *107*, 411–429.

Wüst, S., Van Rossum, E. F., Federenko, I. S., Koper, J. W., Kumsta, R., & Hellhammer, D. H. (2004). Common polymorphisms in the glucocorticoid receptor gene are associated with adrenocortical responses to psychosocial stress. *Journal* *of* *Clinical* *Endocrinology* *and* *Metabolism,* *89,* 565–573.

Attachment**: Manual for the tend-and-befriend training (TBT)**

**Session 1**

Duration: 4h

Goals: Interpersonal communication skills

Documents: Non-disclosure agreement

**Psychoeducation** (30min)

- Function of stress, effects of chronic stress, long-term effects on psychological and somatic health, stress as uncontrollable social evaluation
- Effects of social support on stress, health and wellbeing

**Group cohesion I** (30min)

- Discuss in small groups: What are my hopes and expectations from the course? Collect the inputs, whole group discussion
- Importance of safety in the training, signing of non-disclosure agreement

**Break** (15min)

**Group cohesion II** (60min)

The entire group together builds “Lauf der Dinge” domino like system out of different pieces and parts. The goal is to build a chain of objects that will put each other in movement to cross a few meters distance. The fun and non-competitive task is meant to promote group cohesion. Participants get to know each other in a relaxed way. We make photos that will be shown at the end of training.

**Break** (15min)

**Interpersonal communication skills (60min)**

Good and mindful communication skills are empathy, congruence and unconditional regard of the other. Input from trainers with examples of communication. The effects, the realization and the results of these communication skills are practised in small group exercises and discussed in the whole group.

- Groups of 3-4 participants are formed
- Two participants (Talker and Listener) are communicating, 1 or 2 are observing
- Talker talks about unpleasant experience/situation, listener tries to mindfully listen and to show empathy, be congruent and to show unconditional regard.
- Each communication round has a duration of 15min, all small group members will take turns.
- Observer/s gives feedback on the use of communication skills and elicits emotional results of communication
- In the whole group the difficulties and results of the communication skills are discussed. Also, the importance of this - non-evaluative and affirming - kind of communication to reduce stress is highlighted.

**Discussion** (30min)

In the discussion, the main topics are repeated (good communication matters!) and the individual take home messages are formed. Also, the homework is assigned:

- Homework: Practise the communication skills with on at least two occasions and discuss the difficulties and results until the next training session with one of your training colleagues.

**Session 2**

Duration: 2h

Goals: Experiencing and using feelings

**Psychoeducation** (30min)

- Recapitulation of the previous session, exchange about homework
- Introduction to the importance of being in touch with your feelings for stress management
- Somatic markers and the technique to focus on somatic manifestations of emotions.
- Importance of feeling one owns emotion for interpersonal coping

**Focusing exercise** (30min)

- *Relax, direct attention to somatic sensations*
- *Find one personal problem and find a bodily sensation that is associated with the problem (felt sense);*
- *Felt sense: Give a name/ a description to the feeling*
- *Go back and forth from the sensation to the word and check if the description feels right /if they resonate with each other;*
- *Ask yourself what is it about this problem that makes the quality you just named/pictured*
- *Wait for the answer to come, do not accept the first rationalization coming to your mind, until there is a slight shift in the feeling.*
- Each participant does it for herself and reflects on the experience.

**Break** (15min)

**Using felt senses in interpersonal communication**

- *Form dyads*
- *Express felt sense to listener, who listens with learned communication skills from session 1*
- *Exchange roles*

**Discussion** (15min)

In the discussion, the main topics are repeated (felt sense and interpersonal communication) and the individual take home messages are formed. Also, the homework is assigned

- Homework: Practise the focusing exercise and use felt senses for interpersonal communication

**Session 3**

Duration: 2h

Goal: Social support networks

**Psychoeducation** (15min)

- Recapitulation of the previous session, exchange about homework
- Evidence from selected studies about social support and social network.

**Social support network exercise I** (30min)

- *Form dyads*
- *Participants reconstruct their social support networks out of toy figures, final composition is captured by photo*
- *Status and strength of social ties are depicted by grouping of toy figures and their colours (green, red, blue)*
- *Questions and hints:*

*Does this map represent everybody important in my life? (If forgot somebody important, note it!)*

*Who are the most important connections for me?*

*Do I make use of these resources?*

*Is somebody missing, do I wish/should I build up more connections?*

*How do I feel that I’m supported?*

*Who gives me the most support?*

*Whom do I give the most support?*

*How many types of relationships I have on this map?*

*How many different social roles do I have?*

*Are there connections, which I want to strengthen/revive?*

*Are there connections I’d better weaken/ cut off?*

**Break** (15min)

**Social network exercise I** (30min)

- *Form triads*
- *Discuss feeling when examining your social network*
- *Which social tie/ties could be improved, which should be attenuated*
- *Differential social support: What support from whom?*

**Discussion** (15min)

In the discussion, the main topics are repeated (Know, use and improve your social network) and the individual take home messages are formed. Also, the homework is assigned:

- Homework: *Choose a relationship from your map (take a toy representing this person home) in which you want to change something. Do some actual steps towards this change in real life.*

**Session 4**

Duration: 2h

Goal: Sociometer: Rejection, withdrawal and vulnerability

**Psychoeducation** (15min)

- Self-esteem is a function of social acceptance and interpersonal relationships
- Interpersonal rejection reduces self-esteem and thus has a profound impact on well-being and overall functioning.
- Social recompensation needs talking about feeling rejected

**Sociometer exercise I** (30min)

- *Form dyads*
- *Participants reconstruct recent social rejections and how this made them feel and behave*
- *Elicit emotions during and after social rejection and exclusion*

**Break** (15min)

**Sociometer exercise II** (30min)

- *Form triads*
- *Discuss how it felt to talk about the social rejection and its consequences with another person*
- *How much vulnerability is needed to overcome being vulnerable?*

**Break** (15min)

**Discussion** (15min)

In the discussion, the main topics are repeated (Rejection and vulnerability) and the individual take home messages are formed. Also, the homework is assigned:

- Homework: *Practice being more open and more vulnerable with your surrounding. Call a training partner to exchange about these experiences.*

**Session 5**

Duration: 2h

Goal: Acute stress

**Psychoeducation** (15min)

- Stress is the result of perceived social evaluation
- Acute stress leads to acute stress responses

**Acute stress exercise** (30min)

- *Form triads*
- *Why am I stressed in certain situations?*
- *How do I respond to acute stress in the short and long run?*
- *How do others respond to me being acutely stressed?*

**Break** (15min)

**Inner support team exercise** (30min)

- *Stay in triad*
- *Who of your social support network is in your inner support team?*
- *Role-plays with inner teams. A person selects actors out of other participants play the characters of her inner team and instructs them to their role. Picking some difficult situation where the inner team members will come to your aid.*

**Break** (15min)

**Discussion** (15min)

In the discussion, the main topics are repeated (Acute stress and inner support team) and the individual take home messages are formed. Also, the homework is assigned:

- Homework: *Practice using your inner support team more often.*

**Session6**

Duration: 4h

Goal: Recapitulation

**There and back again** (90min)

- Form triads
- Take a walk for 90 min
- Each participants has 30min to tell the other how he perceived the training and what has changed during the training

**Repeat** (60min)

- Trainers repeat all sessions
- Trainers use photos from all sessions

**Break** (15min)

**Tool box** (30min)

- Form triads
- Participants discuss and formulate their toolbox

**Expressing gratitude** (30min)

- Participants select other participants, whom they want to thank for their support

**Feedback and good bye** (15min)

- Participants and trainers give their feedback and say good bye to each other
